# High expression of SOX10 is correlated with poor prognosis and immune infiltrates in skin cutaneous melanoma

**DOI:** 10.3389/fonc.2025.1444670

**Published:** 2025-04-24

**Authors:** Xu Sun, Yi-ming Cheng, Meng-wei Sun, Xu-dong Zhang, Xiao-yu Yu, Hai-bo Wang, Yi-fei Wang, Ning Li

**Affiliations:** ^1^ Department of Oral and Maxillofacial Surgery, School and Hospital of Stomatology, Hebei Medical University & Hebei Key Laboratory of Stomatology & Hebei Clinical Research Center for Oral Diseases, Shijiazhuang, China; ^2^ Department of Periodontics, School and Hospital of Stomatology, Hebei Medical University & Hebei Key Laboratory of Stomatology & Hebei Clinical Research Center for Oral Diseases, Shijiazhuang, China; ^3^ Department of Prosthodontics, School and Hospital of Stomatology, Hebei Medical University & Hebei Key Laboratory of Stomatology & Hebei Clinical Research Center for Oral Diseases, Shijiazhuang, China

**Keywords:** skin cutaneous melanoma, SOX10, prognosis, immune infiltration, immune checkpoints

## Abstract

**Background:**

Skin Cutaneous Melanoma (SKCM) is a malignant tumor and the prediction of its prognosis remains challenging. Sex determining region Y-box 10 (SOX10) is over-expressed in SKCM and reported to accelerate tumor invasion and immunosuppression. Although studies have suggested the correlation of immune infiltration between SOX10 and SKCM, further in-depth explore of the immunomodulatory role of SOX10 is still needed. Therefore, we assessed the prognostic role of SOX10 and its correlation with immune infiltration and checkpoint expression.

**Methods:**

RNA sequencing data were obtained for analysis of SOX10 expression and differentially expressed genes (DEGs) from the Cancer Genome Atlas (TCGA). Moreover, functional enrichment analysis of SOX10-related DEGs was performed by GO/KEGG, GSEA. Receiver operating characteristic (ROC) curves were used to assess the diagnostic value of SOX10 in SKCM. Kaplan-Meier method was conducted to assess the effect of SOX10 on survival. Additionally, the clinical significance of SOX10 in SKCM was figured out by LASSO and prognostic nomogram model. We analyzed SOX10-related immune cell infiltration and expression of immune checkpoints. Finally, validations were performed through immunohistochemical analysis.

**Results:**

SOX10 was low expressed in a range of malignant tumor tissues except SKCM. Totally, 1029 differentially significant genes (DSGs) were identified between SOX10 low- and high- expression group, of which 50 genes were upregulated and 979 genes were downregulated. Additionally, SOX10 high expression was remarkably associated with pathologic stage, age and breslow depth in a sample of 472 cases (P *<* 0.05). Screening was performed by LASSO coefficients to select non-zero variables that satisfied the coefficients of lambda, and 8 genes were screened out. The forest plot results showed that only OCA2 and TRAT1 had statistical significance (P < 0.05) by multi-factor COX regression analysis. SOX10, OCA2, TRAT1, pathologic stage, age and breslow depth were included in the nomogram prognostic model. Furthermore, upregulation of SOX10 expression inhibited immune infiltration in SKCM.

**Conclusion:**

Overall, high expression of SOX10 was correlated with poor prognosis in SKCM, which may be related to suppression of immune infiltration. The DSGs and pathways identified in our research have initially provided an insight into the molecular mechanisms underlying the progression of SKCM.

## Background

SKCM is one of the malignant tumors of skin caused by excessive proliferation of abnormal melanomas (1), which can also occur in the mucous and viscera, with fast progression and invasion, poor prognosis and increased mortality rate (2). The incidence rate of disease in China was nearly 0.9/100000, and about 20000 new patients each year (3). SKCM often occurs on the basis of pigmented nevi, mainly due to malignant transformation of junction or mixed nevi (4), diagnosis of which mainly depends on imaging and histopathology examination. The treatment of early and advanced patients relies primarily on radiation therapy and immunization therapy, respectively (5–7). Standardized diagnostic and therapeutic procedures are still lacking in clinical practice, due to the complexity of the pathological mechanisms. The clinical requirements of patients need to be met urgently (8, 9). Therefore, it is significant to explore more sensitive biomarkers for SKCM diagnosis and targeted therapy.

SOX is a class of transcription factors belonging to the high mobility group protein (HMG), which present in animals widely, with 20 subtypes identified in mammals (10, 11). SOX10 belongs to the E subgroup of this family and is crucial for the growth and development in nerve cells (12). Serum SOX10 has been reported to reflect the status of SKCM patients after therapy, providing a timely assessment of clinical benefit (13). High level of SOX10 in peripheral blood suggested a relatively short cell cycle or melanocyte death, which can be induced by T cell (14). In addition, SOX10 could be a diagnostic marker for metastatic melanoma in sentinel lymph nodes (15). Recently, it has been shown that SOX10/SOX11/MITF (melanocyte inducing transcription factor) can be taken as a diagnostic and therapeutic indicator for SKCM (16). Studie have confirmed that loss of SOX10 reduces proliferation, leads to invasive properties, including the expression of mesenchymal genes and extracellular matrix, and promotes tolerance to BRAF and/or MEK inhibitors (17). There are also studies confirm that SOX10 hinders immunogenicity of melanoma cells through the IRF4-IRF1 axis (18). Other scholars have discovered that Sox10 knockout effects on tumor growth on CD8^+^ T cells, which is a negative correlation with SOX10 and immune-related pathways (19). So further research is still needed on the immune regulatory mechanism of SOX10 in SKCM.

Therefore, this research aimed to determine the correlation between expression of SOX10 and prognosis of SKCM. First of all, RNA-seq data of SKCM from TCGA were acquired to analyze the expression of SOX10. Moreover, functional enrichment analysis of SOX10 related DEGs was performed via GO, KEGG, GSEA. We further evaluated the diagnostic and prognostic values, the correlation with immune infiltrates and immune checkpoints of SOX10 in SKCM. Our study links the overexpression of SOX10 and poor survival in SKCM. In this manner, remarkably altered genes and pathways will be screened, the connection of which with SOX10 might play key roles in SKCM.

## Methods

### Tissue-specific expression of SOX10

Tissue expression levels of SOX10 were retrieved using the *Human Protein Atlas (HPA)* (https://www.proteinatlas.org/) database.

### Expression of SOX10

Detection of SOX10 expression in pan-cancer, SKCM and pathological stages of SKCM using the *GEPIA* (http://gepia.cancer-pku.cn/index.html) database.

### Sample collection

This study was conducted in accordance with the ethical principles of the Declaration of Helsinki. Medical research involving human subjects or data was approved by the Ethics Committee of the Hospital of Stomatology of Hebei Medical University (Shijiazhuang, China)(No.[2018]019). 8 samples of SKCM and 4 samples of paracancerous tissue were collected from the patients’ cheek and lip for immu-nohistochemical experiments at the Department of Oral and Maxillofacial Surgery, Hospital of Stomatology, Hebei Medical University between 2010 and 2024. Samples of paracancerous tissue were taken from an area 1-1.5 cm outside the tumor border obtained by extended dresection of SKCM. Informed consent was obtained from all patients or their relatives. **Supplementary Table** summarizes the patients’ demographic characteristics.

### Expression and enrichment analysis of SOX10 correlated genes

*LinkedOmics* (http://www.linkedomics.org/) (20) was used to screen positively and negatively related genes with SOX10. Related genes with the adjusted P-value < 0.05 were applied for GO&KEGG analysis using R package ggplot (21).

### Differentially expressed genes analysis

R package limma was adopted to compare expression data of low- and high-expression of SOX10 (cut-off value of 50%) in SKCM samples to identify DEGs (22) Volcano plots was used to visualize the result.

### Functional enrichment analysis

DEGs with the threshold for |log fold change (logFC)| > 1.5 and adjusted P-value (adj P) < 0.05 were applied for functional enrichment analysis. A ggplot2 package was used to map the Gene ontology(GO) and Kyoto Encyclopedia of Genes and Genomes(KEGG) pathways of DEGs using R.

### Gene set enrichment analysis

R package ClusteProfiler (3.14.3) was used for GSEA to elucidate the functional and pathway differences between the high- and low-expression groups of SOX10 (21). The gene set was permutated 1,000 times for each analysis. Adjusted P-value < 0.001 and FDR q-value < 0.001 were considered to be statistically significant.

### Correlation analyses for SOX10 expression and clinicopathological characteristics of SKCM patients

Baseline characteristics were compared for high- and low- expression of SOX10 groups using the Wilcoxon rank sum test (continuous variables) or Spearman chi-square test (rank variables). The correlation between SOX10 and clinicopathological characteristics was evaluated by univariate logistic analysis. Diagnostic value of SOX10 was demonstrated by ROC curves in pathologic stages, age and breslow depth. R package limma was used to analyze the differences in expression of SOX10 among the aforementioned subgroups.

### Prognostic model generation and calibration

Patients were divided into high- and low- groups based on the median value of SOX10 expression. KM curves were performed by survival package (version 3.2-10). Glmnet package (version 4.1-2) & survival package (version 3.2-10) were used for LASSO coefficient filtering. Select non-zero variables that satisfy the coefficients of lambda.min. Risk characteristics were constructed by further screening to determine the final SOX10-related genes. The risk score was calculated as follows risk score = Σ(expression level of each gene × correlation coefficient). Multivariate COX regression coefficients based on prognosis-associated genes.

In order to individualize the prediction of overall survival (OS) in SKCM patients, a nomogram was generated using the RMS R package (version 5.1-3), which included genes screened by LASSO coefficient filtering and calibration plots. The calibration curves were evaluated graphically by mapping the nomogram-predicted probabilities against the observed rates, and the 45°line represented the best predictive values. Concordance index (C-index) was used to determine the discrimination of the nomogram, and the bootstrap approach was used to calculate 1000 resamples. All statistical tests were double tailed with 0.05 as the statistical significance level.

### Immune infiltration analysis

The ssGSEA package and ESTIMATE package were used for immuno-infiltration correlation analysis of SOX10. The correlation between SOX10 expression and immune cells infiltration was evaluated by Spearman chi-square test.

### Immune checkpoints expression analysis

R package limma was used to detect the expression differences of various immune checkpoints. The correlation between SOX10 expression and immune checkpoints expression was evaluated by Spearman chi-square test.

### Immunohistochemistry validation and human protein atlas database

We investigated the protein expression levels of the SOX10 signature in SKCM samples by using the HPA database (https://www.proteinatlas.org/) and validated the findings by immunohistochemical experiments, as described below 8 SKCM tissue samples and 4 paracancerous tissue samples were cut into 4μm slices, fixed in 10% formalin, and embedded in paraffin. Citrate buffer was used for antigen retrieval, Protein expression was assessed using the PV9000 two-step method and the Poly HRP anti-mouse/rabbit IgG detection system (Zhongshan Jingiao Biotechnology Company, Beijing, China). All procedures were performed according to the manufacturer’s instructions. Color development was assessed using 3,3’-diaminobenzidine (Zhongshan Jingiao Biotechnology Company, Beijing, China). Counterstaining was performed using hematoxylin-eosin. Routine dehydration and the final mounting were performed. The anti-SOX10 primary antibody (dilution 1:100) was purchased from Servicebio (Wuhan, China) and diluted with phosphate-buffered saline. The sections were incubated with the primary antibody at 4°C overnight, followed by incubation with the secondary antibody (HRP-conjugated goat anti-rabbit, 100 μL) at room temperature for 20 minutes. Chromogenic detection was performed using diaminobenzidine (DAB), after which the sections were dehydrated and counterstained with hematoxylin. Microscopic examination and image acquisition were subsequently conducted. Hematoxylin stained the nuclei blue, while DAB-positive expression was visualized as brown-yellow. The intensity of the positive staining correlated with the antigen content and distribution density, with stronger staining indicating higher antigen levels. The staining results were categorized as follows: blue, negative; light yellow, weakly positive; brown-yellow, moderately positive; and dark brown, strongly positive.

### Comparison of tumor volume in tumor-bearing mice: animal experiment

To establish SKCM tumor models in wild-type and SOX10^+/+^ mice, tumor volume and survival were compared. The experimental procedure is described as follows: C57BL6J mice were randomly divided into two groups, with five mice in each group. The dorsal skin of the mice was shaved in a central area of approximately 2*2 cm and disinfected with 75% ethanol. The SOX10^+/+^ group was injected with 0.2 ml of BRAF^V600E^CDKN2A^−/−^SOX10^+/+^ cells at a concentration of 4*10^6^/ml, while the wild-type group was injected with 0.2 ml of BRAF^V600E^CDKN2A^−/−^ cells at the same concentration. Starting from the day of injection, the mice were monitored daily for food intake and activity. The day when all mice in the experimental groups developed palpable black nodules approximately the size of mung beans on their backs was designated as the tumor onset day. Tumor length (a), width (b), and height (c) were measured every four days, and tumor volume was calculated using the formula V = abc (mm³). Tumor growth curves were plotted accordingly. The experiment was terminated when 80% of the mice in the experimental groups exhibited signs of lethargy. Tumor volumes were measured and recorded on days D4, D8, D12, D16, and D20, and tumor growth curves were plotted for both groups.

### Statistical analysis

All statistical analyses were performed with R (V 3.6.3). The expression of SOX10 in unpaired samples was analyzed by Wilcoxon rank-sum test. Logistic regression analysis was used to evaluate the relationship between clinical characteristics and SOX10 expression. Cox regression analysis and Kaplan-Meier method were used to evaluate the prognostic factors. Multivariate Cox analysis was adopted to compare the impact of SOX10 related genes. In all tests, P < 0.05 was considered statistically significant.

## Results

### Expression of SOX10 in pan-cancers and SKCM

**Figure 1:**
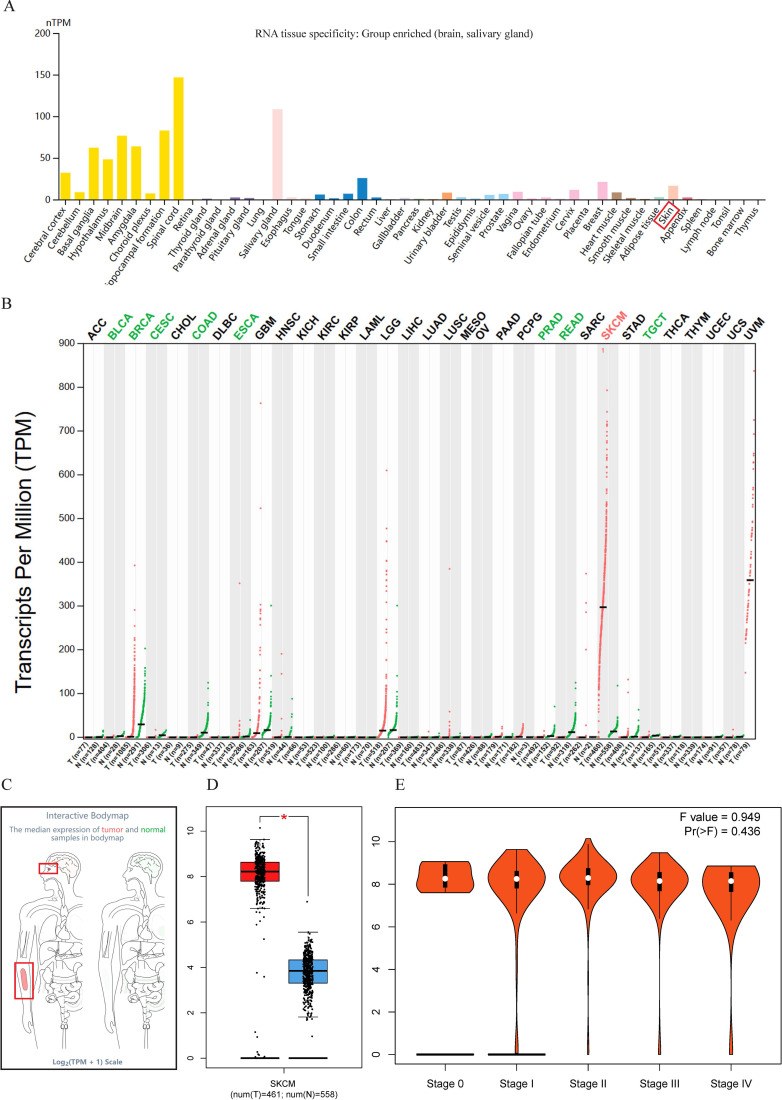
Expression profile of SOX10. **(A)** Tissue-specific expression of SOX10; **(B)** Expression of SOX10 in pan-cancer; **(C)** Expression of SOX10 in the interactive body map; **(D)** Expression of SOX10 in SKCM; **(E)** SOX10 expression in pathological stages of SKCM.

There is no tissue specificity for SOX10 expression, except brain and salivary gland tissue (**Figure 1A**). Analysis of SOX10 expression in pan-cancer tissues by *GEPIA* revealed that SOX10 was significantly highly expressed in SKCM (**Figures 1B, D**). SOX10 was highly expressed in the skin and eye in the interactive body map (**Figure 1C**), in accordance with the preferred areas of tumor. Notably, no significant difference of SOX10 expression was observed across pathologic stages (**Figure 1E**).

### Enrichment analysis of SOX10 and co-expressed genes in SKCM

**Figure 2:**
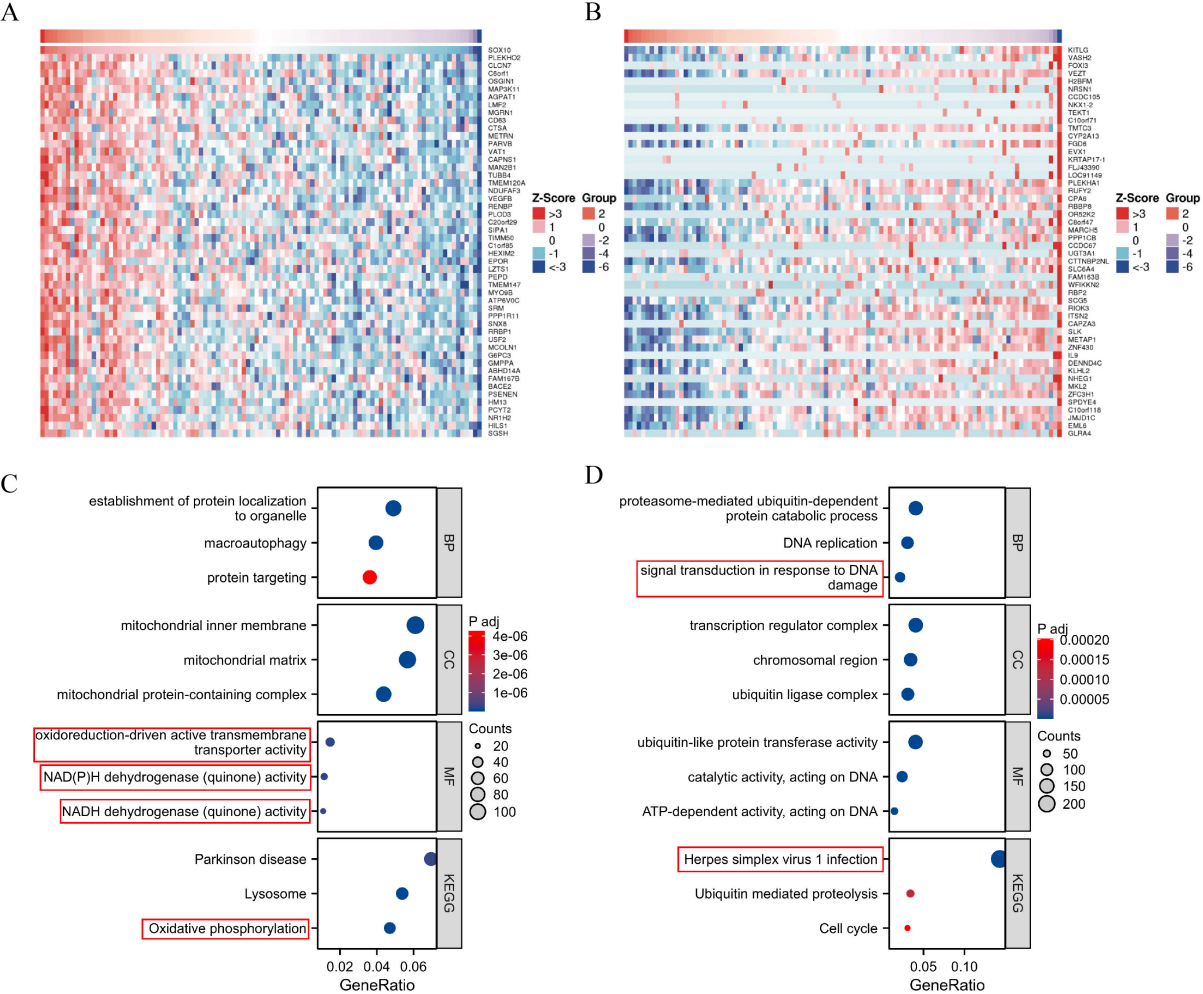
Enrichment analysis of SOX10 and co-expressed genes in SKCM. **(A)** Heatmap of the top 50 positively related genes; **(B)** Heatmap of the top 50 negatively related genes; **(C)** GO and KEGG terms of positively related genes; **(D)** GO and KEGG pathway terms of negatively related genes.

Heatmaps of the top 50 positively/negatively SOX10 related genes were shown in **Figures 2A, B**. Functional annotations indicated these positively related genes were involved in “oxidoreduction-driven active transmembrane transporter activity”, “NAD(P)H dehydrogenase (quinone) activity”, “NADH dehydrogenase (quinone) activity” and “oxidative phosphorylation” (**Figure 2C**), indicating that SOX10 could promote metabolism in SKCM. The enrichment results of negatively related genes were shown in **Figure 2D**.

### Differential expression and enrichment analysis in SKCM with low- and high-expressed SOX10

**Figure 3:**
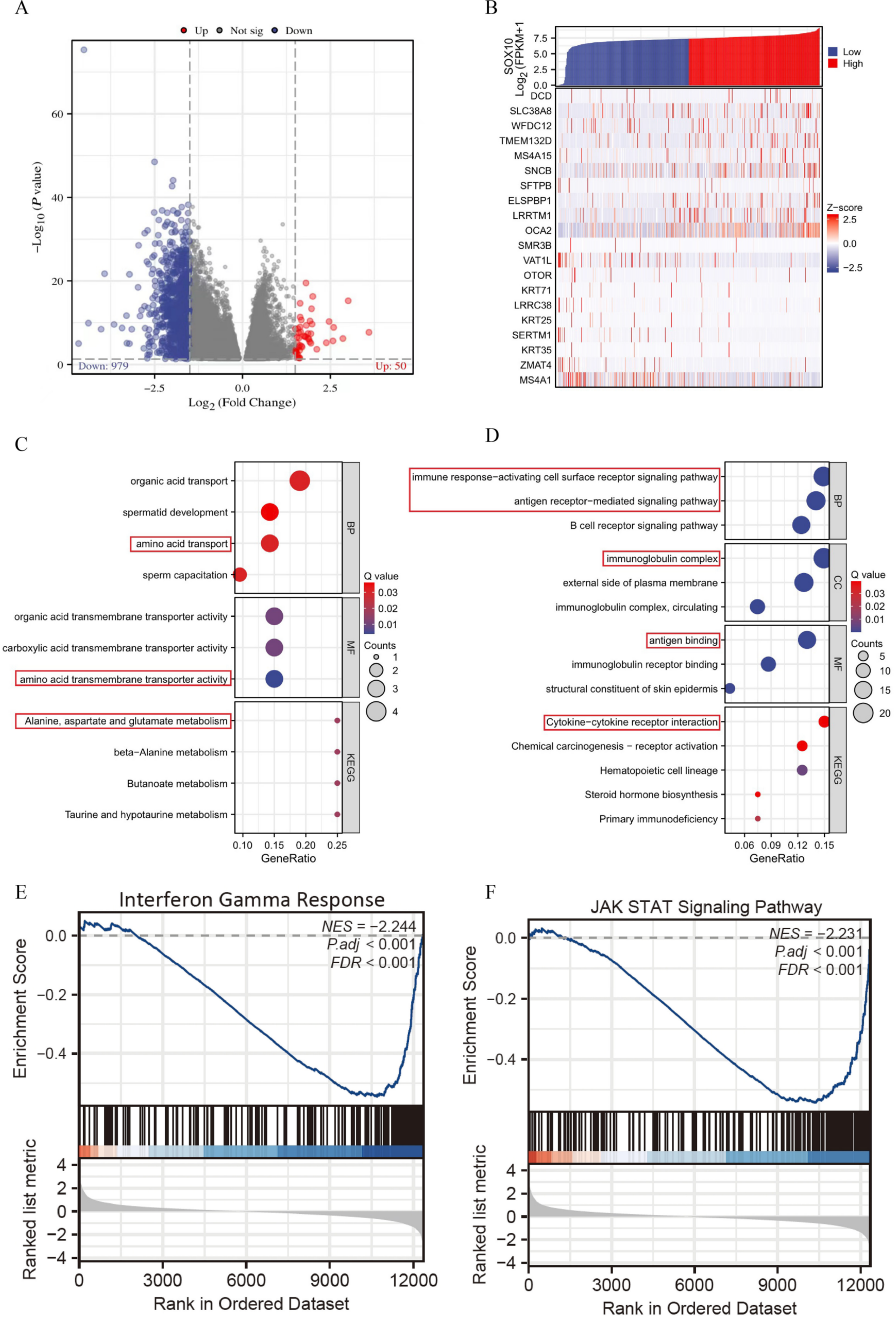
Enrichment analysis of DEGs in SKCM with low- and high-expressed SOX10. **(A)** Volcano plot of DEGs; **(B)** Heatmap of correlation; **(C, D)** GO&KEGG terms of DEGs; **(E, F)** GSEA analysis in low- SOX10 expression datasets.

A total of 1029 DEGs, including 50 up-regulated and 979 down-regulated, were identified between SOX10 low- and high-expressed groups (|logFC| >1.5 and adj P <0.05) (**Figure 3A**). Co-expression heatmaps showed that some genes have consistent expression, while others have opposite expression (**Figure 3B**). Enrichment analysis showed that up-regulated genes were significantly enriched in terms of “amino acid transport” (BP), “amino acid transmembrane transporter activity” (MF), “alanine, aspartate and glutamate metabolism” (KEGG) (**Figure 3C**). Down-regulated genes showed significant enrichment of “immune response-activating cell surface receptor signaling pathway” (BP), “antigen receptor-mediated signaling pathway” (BP), “immunoglobulin complex” (CC), “antigen binding” (MF) and “cytokine-cytokine receptor interaction” (KEGG) (**Figure 3D**). To further understand the biologic pathways involved in SKCM with SOX10 expression, GSEA was performed to identify critical signaling pathways. Significant differences (FDR <0.001, adj P <0.001) were observed in the enrichment of significantly different genes. Interferon Gamma Response and JAK STAT3 signaling were presented significantly enriched in SOX10 low-expression phenotype (**Figures 3E–F**).

### Association between SOX10 expression and clinical features of SKCM

**Figure 4:**
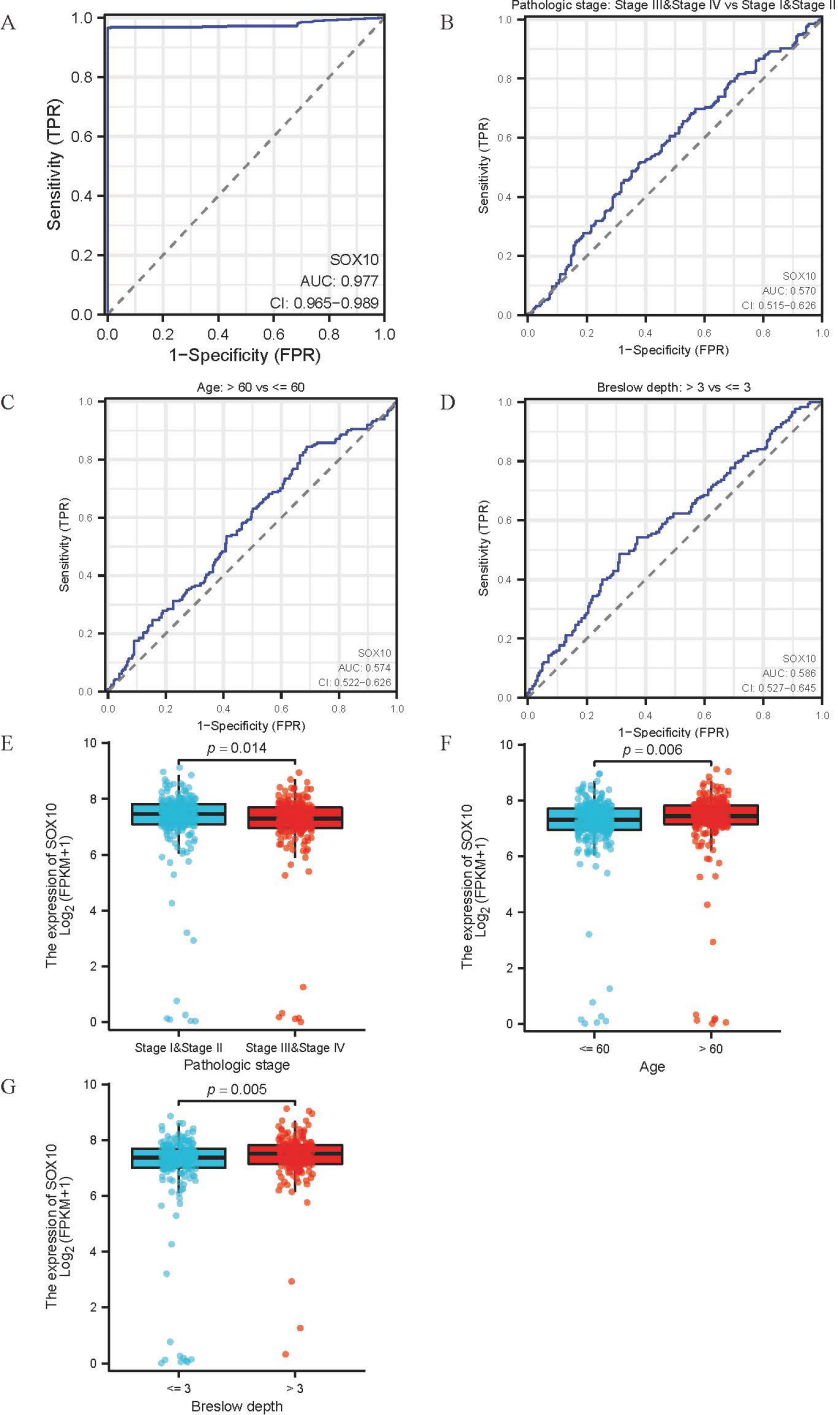
Association between SOX10 expression and clinical features of SKCM. **(A-D)** ROC curve for diagnostic efficacy of SOX10 in all SKCM patients and subsets (pathologic stages, age and breslow depth); **(E-G)** Differential expression of SOX10 in subsets (pathologic stages, age and breslow depth).

The main baseline characteristics of SKCM in TCGA was shown in **Table 1**. The results indicated that SOX10 was significantly correlated with pathologic stage, age and breslow depth (P < 0.05). Logistic analysis was applied to further verify the relationship between SOX10 and SKCM clinical characteristics (**Table 2**). As a result, SOX10 was an independent risk factor in these subgroups of patients, including pathologic stage I & II (Odds Ratio [OR], 0.673; P < 0.05), age > 60 (OR, 1.523; P < 0.05), and breslow depth > 3 cm (OR, 1.667; P < 0.05). Furthermore, SOX10 could have diagnostic value in SKCM patients as well as in the subgroups of patients mentioned above, as revealed by ROC curves, with the AUC of 0.977, 0.570, 0.574 and 0.586 respectively (**Figures 4A–D**). Besides, the Wilcoxon Rank SUM test was used to compare the expression of SOX10 in patients with different clinicopathological features. The result showed that SOX10 was significantly high-expressed in pathologic stage I & II, age>60, breslow depth > 3cm patients (P < 0.05) (**Figures 4E–G**), which indicated that SOX10 might be a potential biomarker in SKCM.

**Table 1:** Baseline data.

Characteristics	Low expression of SOX10	High expression of SOX10	P value
n	236	236	
Pathologic T stage, n (%)			0.363
T1	21 (5.8%)	21 (5.8%)	
T2	41 (11.2%)	38 (10.4%)	
T3	45 (12.3%)	46 (12.6%)	
T4	63 (17.3%)	90 (24.7%)	
Pathologic N stage, n (%)			0.301
N0	108 (26%)	128 (30.8%)	
N1	38 (9.2%)	36 (8.7%)	
N2	26 (6.3%)	23 (5.5%)	
N3	33 (8%)	23 (5.5%)	
Pathologic M stage, n (%)			0.892
M0	207 (46.6%)	212 (47.7%)	
M1	12 (2.7%)	13 (2.9%)	
Pathologic stage, n (%)			0.128
Stage I	39 (9.4%)	39 (9.4%)	
Stage II	58 (14%)	82 (19.9%)	
Stage III	94 (22.8%)	77 (18.6%)	
Stage IV	12 (2.9%)	12 (2.9%)	
Gender, n (%)			0.850
Female	91 (19.3%)	89 (18.9%)	
Male	145 (30.7%)	147 (31.1%)	
Race, n (%)			0.885
Asian	6 (1.3%)	6 (1.3%)	
Black or African American	1 (0.2%)	0 (0%)	
White	223 (48.3%)	226 (48.9%)	
**Age, n (%)**			**0.025**
<= 60	138 (29.7%)	115 (24.8%)	
> 60	93 (20%)	118 (25.4%)	
Weight, n (%)			0.117
<= 70	32 (12.4%)	45 (17.4%)	
> 70	95 (36.7%)	87 (33.6%)	
Height, n (%)			0.774
< 170	56 (22%)	62 (24.4%)	
>= 170	67 (26.4%)	69 (27.2%)	
BMI, n (%)			0.141
<= 25	35 (13.9%)	49 (19.5%)	
> 25	86 (34.3%)	81 (32.3%)	
Tumor tissue site, n (%)			0.189
Extremities	93 (22.1%)	105 (25%)	
Trunk	78 (18.6%)	93 (22.1%)	
Head and Neck	18 (4.3%)	20 (4.8%)	
Other	10 (2.4%)	3 (0.7%)	
Melanoma ulceration, n (%)			0.154
No	73 (23.2%)	75 (23.8%)	
Yes	69 (21.9%)	98 (31.1%)	
Melanoma Clark level, n (%)			0.853
I&II	13 (4%)	11 (3.4%)	
III	37 (11.5%)	41 (12.7%)	
IV	80 (24.8%)	88 (27.2%)	
V	23 (7.1%)	30 (9.3%)	
**Breslow depth, n (%)**			**0.016**
<= 3	99 (27.4%)	87 (24.1%)	
> 3	71 (19.7%)	104 (28.8%)	
Radiation therapy, n (%)			0.527
No	189 (40.6%)	195 (41.9%)	
Yes	43 (9.2%)	38 (8.2%)	

The values in bold denote that the main baseline characteristics of SKCM in TCGA indicated that SOX10 was significantly correlated with pathologic stage, age, and breslow depth (P < 0.05).

**Table 2:** Univariate logistic regression.

Characteristics	Total (N)	OR (95% CI)	P value
Pathologic T stage (T3&T4 vs. T1&T2)	365	1.323 (0.855 - 2.048)	0.209
Pathologic N stage (N2&N3 vs. N0&N1)	415	0.694 (0.445 - 1.084)	0.108
Pathologic M stage (M1 vs. M0)	444	1.058 (0.472 - 2.372)	0.892
**Pathologic stage (Stage III&Stage IV vs. Stage I&Stage II)**	**413**	**0.673 (0.457 - 0.992)**	**0.046**
Gender (Male vs. Female)	472	1.037 (0.715 - 1.503)	0.850
Race (Black or African American&White vs. Asian)	462	1.009 (0.321 - 3.175)	0.988
**Age (> 60 vs. <= 60)**	**464**	**1.523 (1.054 - 2.199)**	**0.025**
Weight (> 70 vs. <= 70)	259	0.651 (0.380 - 1.116)	0.119
Height (>= 170 vs. < 170)	254	0.930 (0.568 - 1.524)	0.774
BMI (> 25 vs. <= 25)	251	0.673 (0.396 - 1.142)	0.142
Tumor tissue site (Trunk&Head and Neck&Other vs. Extremities)	420	0.969 (0.660 - 1.423)	0.873
Melanoma ulceration (Yes vs. No)	315	1.382 (0.885 - 2.159)	0.154
Melanoma Clark level (IV&V vs. I&II&III)	323	1.102 (0.689 - 1.762)	0.686
**Breslow depth (> 3 vs. <= 3)**	**361**	**1.667 (1.098 - 2.530)**	**0.016**
Radiation therapy (Yes vs. No)	465	0.857 (0.530 - 1.384)	0.527

Values in bold denote that SOX10 was an independent risk factor in these subgroups of patients, including pathologic stages I and II [Odds Ratio (OR), 0.673; P < 0.05], age > 60 (OR, 1.523; P < 0.05), and breslow depth > 3 cm (OR, 1.667; P < 0.05).

### The prognosis analysis of SOX10 in SKCM patients

**Figure 5:**
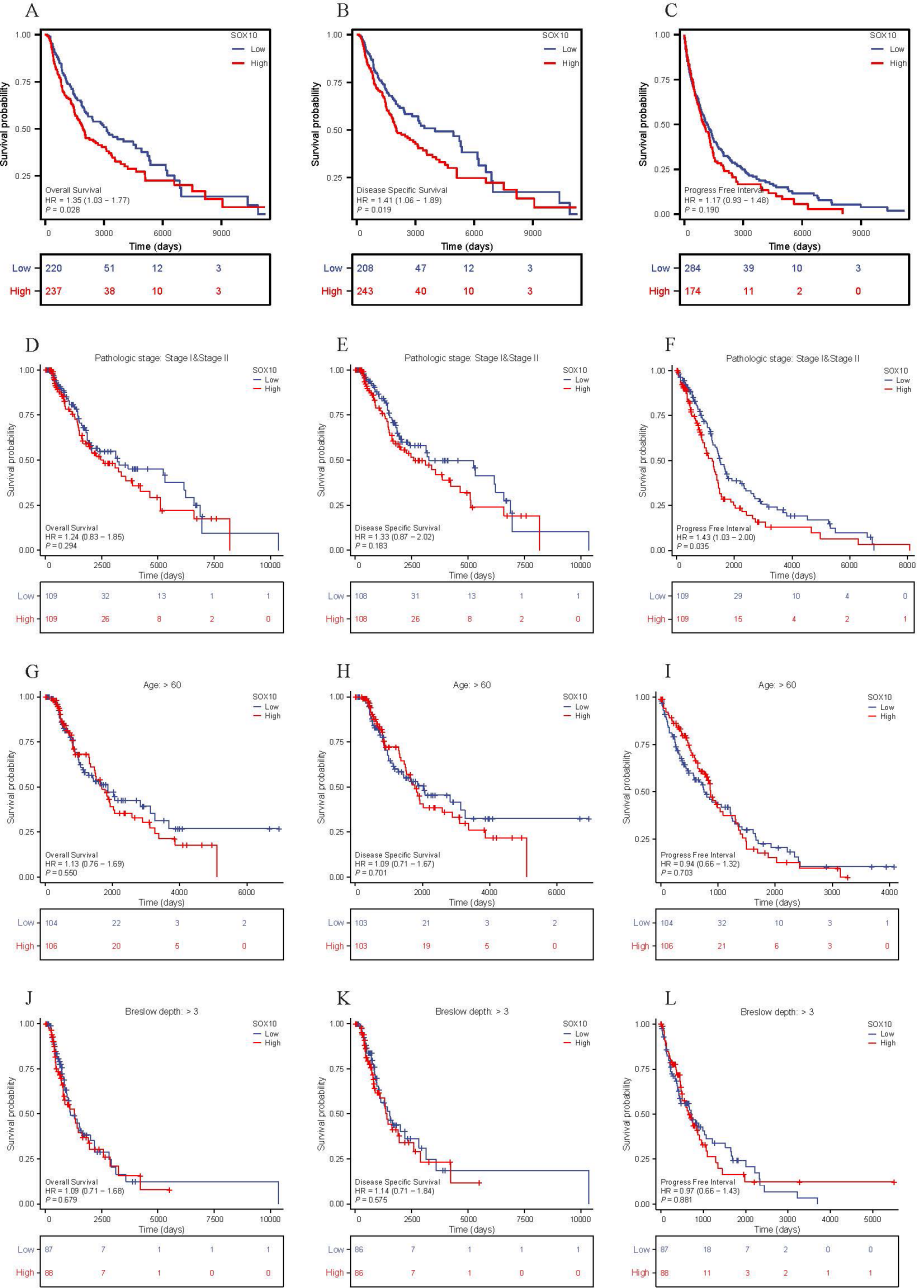
The prognosis value of SOX10 in SKCM. **(A-L)** KM curves in all SKCM patients(OS&DSS&PFI); SKCM patients with pathologic stage I&II, age>60 and breslow depth>3 (OS&DSS&PFI).

The relationship between SOX10 expression and prognosis was analyzed in SKCM patients by using Kaplan-Meier. Patients with high expression of SOX10 had a poor prognosis [OS, HR = 1.35 (1.03 – 1.77), P = 0.028; DSS, HR = 1.41 (1.06 − 1.89) P = 0.019, PFI, HR = 1.17 (0.93 − 1.48), P = 0.190] (**Figures 5A–C**). However, SOX10 could not be considered as a prognostic indicator for subgroups of patients, including pathologic stage I & II, age > 60 and breslow depth > 3 (**Figures 5D–L**).

### Prognostic model of SOX10 in SKCM

**Figure 6:**
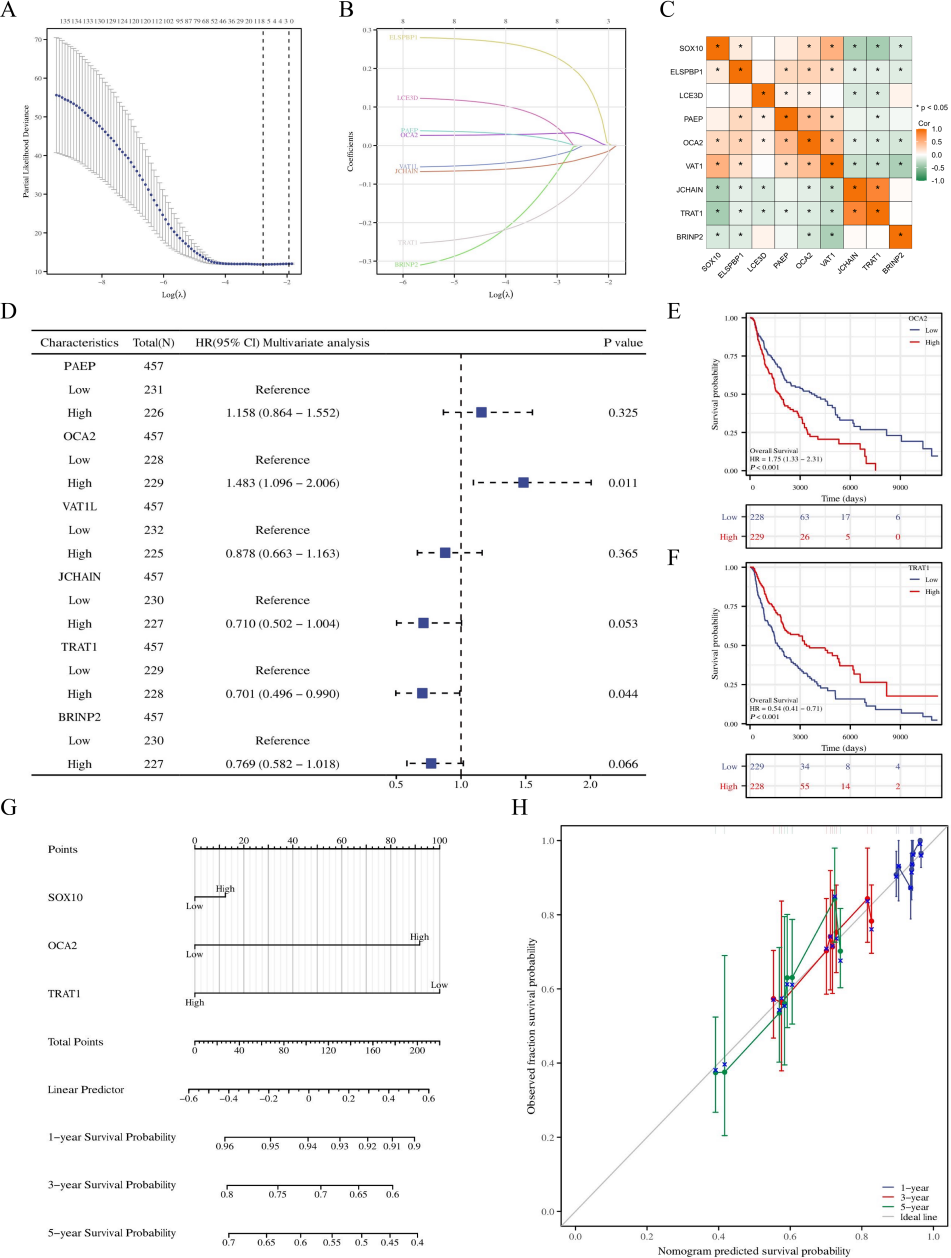
Prognostic model of SOX10 in SKCM. **(A, B)** SOX10 related genes screened by LASSO; **(C)** correlation analysis was performed on SOX10 related genes; **(D)** Forest plot of SOX10 related genes; **(E, F)** KM curves in all SKCM patients with OCA2 and TRAT1 expression; **(G)** Nomogram for predicting the probability of 1-, 3-, 5- year OS for SKCM; **(H)** Calibration plot of the nomogram.

To further explore the prognostic value of SOX10 in SKCM, co-expressed genes of SOX10 were screened by LASSO coefficients, selected non-zero variables by lambda.min. 8 genes related with SOX10 were screened out at last. The risk score = (0.2088*ELSPBP1 expression level, +0.0328*OCA2 expression level, +0.0049*PAEP expression level, +0.018*LCE3D expression level, -0.016*VAT1L expression level, -0.023*BRINP2 expression level, -0.133*TRAT1 expression level, -0.042*JCHAIN expression level) (**Figures 6A, B**). Subsequent correlation analysis was performed on 8 genes and SOX10, revealing that SOX10 positively correlates with ELSPBP1, OCA2, and VAT1 (P < 0.05), and negatively correlates with JCHAIN, TRAT1, and BRINP2 (P < 0.05). (**Figure 6C**) Multifactorial COX regression analysis of these genes was performed by survival R package (**Table 3**) and presented as forest plot (**Figure 6D**). The results showed that OCA2 and TRAT1 were negative prognostic indicators and positive prognostic indicators, respectively, as demonstrated by KM curves (**Figures 6E, F**). Given the effects of OCA2 and TRAT1, a nomogram was constructed including expression of SOX10, OCA2 and TRAT1 (**Figure 6G**). The 1-, 3-, 5-year survival probability was determined by drawing a vertical line downward on the total point axis suggesting the probability of 1-, 3- and 5-year > 50%. The prediction results of the nomogram calibration curve of OS were consistent with all patients’ observation results (**Figure 6H**). Combined with the results of survival analysis, it suggests that SOX10 can be used as a prognostic indicator for SKCM. In addition, patient survival could be accurately predicted based on SOX10/OCA2/TRAT1 expression levels. We retrieved the dataset (GSE98394) from the GEO database to further validate the model’s accuracy. In the GSE98394 dataset, significant differences were observed in the overall survival (OS) rates between high and low risk score groups for SOX10, OCA2, and TRAT1, P= 0.0027, P=0.0116, P=0.0166 (**Supplementary Figures S1A–C**), The results showed that OCA2 and TRAT1 were negative prognostic indicators and positive prognostic indicators (**Supplementary Figure S1D**) Given the effects of OCA2 and TRAT1, a nomogram was constructed including expression of SOX10, OCA2 and TRAT1. The 1-, 3-, 5-year survival probability was determined by drawing a vertical line downward on the total point axis suggesting the probability of 1-, 3- and 5-year > 50% (**Supplementary Figure S1E**). The predictive model demonstrates high accuracy in the validation cohort, and its trends align with the predictions derived from the TCGA database. In conclusion, this model can reliably estimate the overall survival of SKCM patients over 1-, 3- and 5-year.

**Table 3:** Multifactor COX regression of genes screened by LASSO.

Characteristics	Total (N)	Univariate analysis		Multivariate analysis	
		Hazard ratio (95% CI)	P value	Hazard ratio (95% CI)	P value
PAEP	457				
Low	231	Reference		Reference	
High	226	1.397 (1.062 - 1.837)	**0.017**	1.158 (0.864 - 1.552)	0.325
**OCA2**	**457**				
**Low**	**228**	**Reference**		**Reference**	
**High**	**229**	**1.753 (1.331 - 2.307)**	**< 0.001**	**1.483 (1.096 - 2.006)**	**0.011**
VAT1L	457				
Low	232	Reference		Reference	
High	225	0.743 (0.568 - 0.972)	**0.030**	0.878 (0.663 - 1.163)	0.365
JCHAIN	457				
Low	230	Reference		Reference	
High	227	0.544 (0.415 - 0.713)	**< 0.001**	0.710 (0.502 - 1.004)	0.053
**TRAT1**	**457**				
**Low**	**229**	**Reference**		**Reference**	
**High**	**228**	**0.544 (0.414 - 0.714)**	**< 0.001**	**0.701 (0.496 - 0.990)**	**0.044**
BRINP2	457				
Low	230	Reference		Reference	
High	227	0.702 (0.536 - 0.920)	**0.010**	0.769 (0.582 - 1.018)	0.066

Values in bold denote that OCA2 and TRAT1 were negative prognostic indicators and positive prognostic indicators (P < 0.05).

### Immune infiltration analysis in SKCM

**Figure 7:**
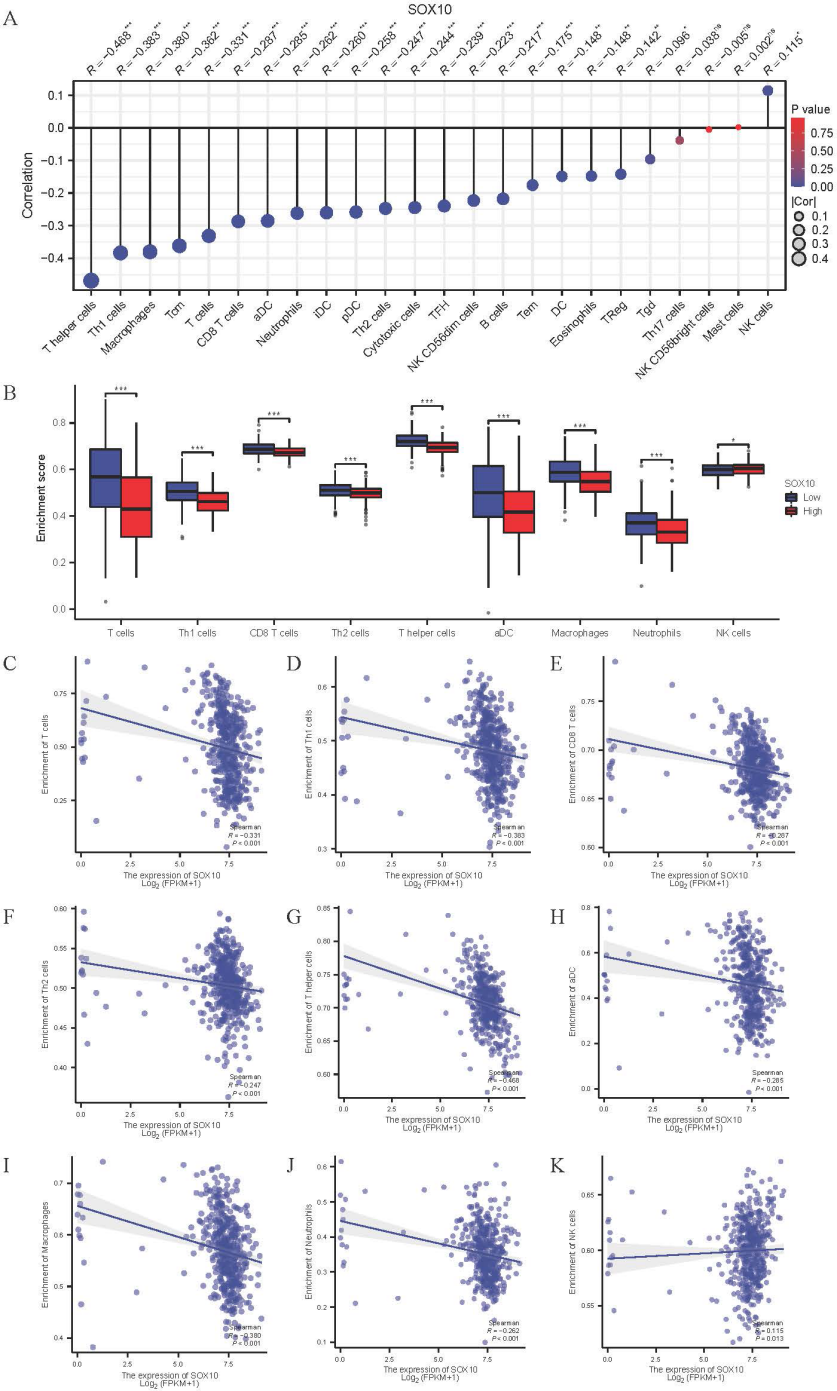
Correlation of SOX10 expression and immune infiltration in SKCM. **(A)** The Lollipop chart of correlation between SOX10 and immune cells. The size of dots showed the absolute value of Spearman R. **(B)** Correlation between the relative enrichment score of aDC, Macrophages, Neutrophils, Th1, Th2, CD8^+^ T cells, T cells, NK cells and the expression level of SOX10; **(C–K)** Infiltration of T cells, Th1, CD8 T cells, Th2, T helper cells, aDC, macrophages, neutrophils, NK cells related to SOX10 expressed tendency.

Immune infiltration plays a key role in tumor progression, which was quantified by ssGSEA. The level of immune cells was negatively correlated with SOX10 expression in SKCM, except for NK cells (**Figures 7A, B**). Spearman correlation analysis was shown in **Figures 7C–K**, suggesting that SOX10 could foster immunosuppressive effects in SKCM.

### Relationship between SOX10 and immune checkpoints in SKCM

**Figure 8:**
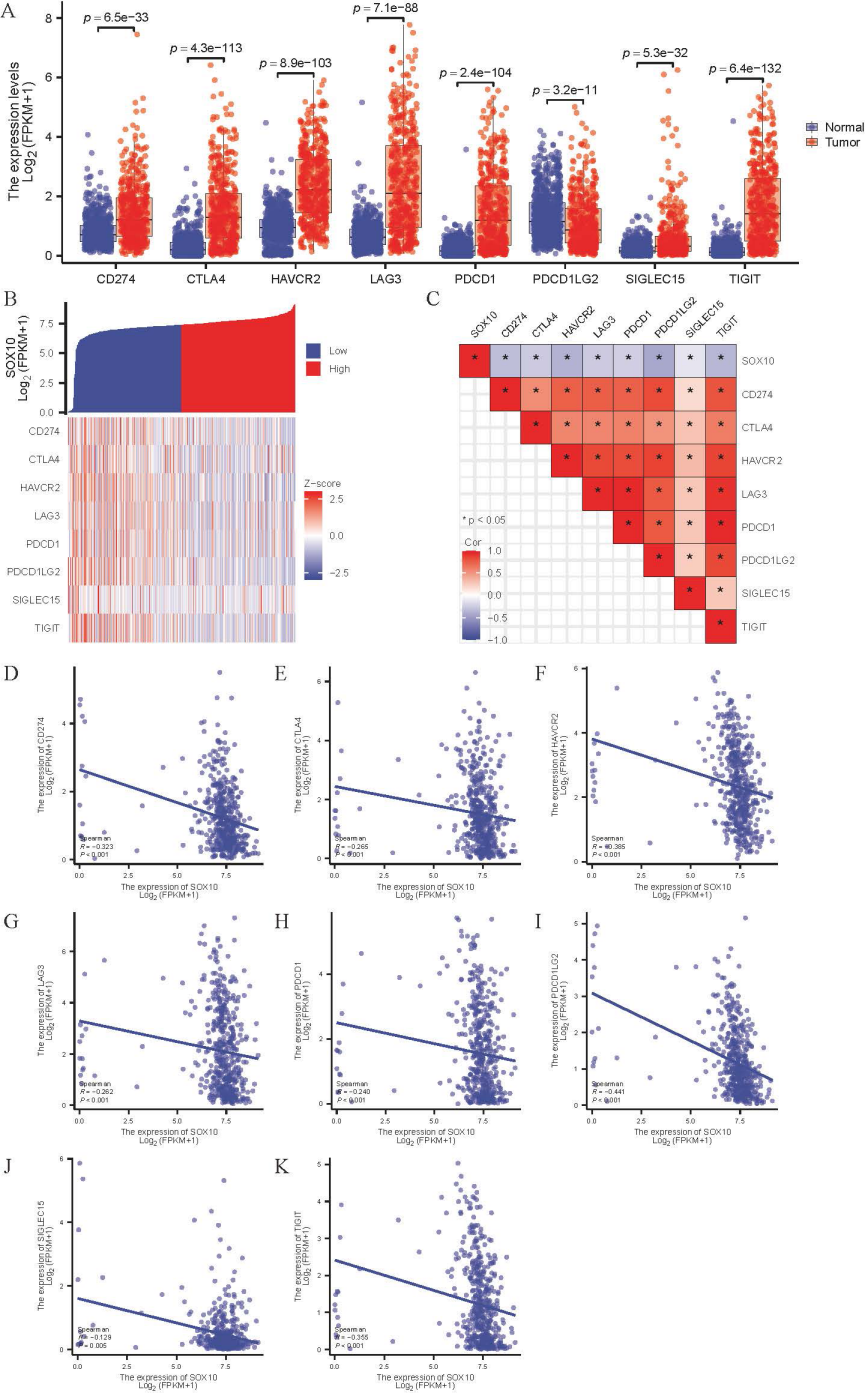
Correlation of SOX10 expression and immune checkpoints in SKCM. **(A)** Expression of immune checkpoints in SKCM; **(B, C)** Heatmap of co-expression and correlation between SOX10 and immune checkpoints; **(D-K)** Expression correlation between CD274, CTLA4, HAVCR2, LAG3, PDCD1, PDCD1LG2, SIGLEC15, TIGIT and SOX10.

Various immune checkpoints are highly expressed in SKCM (**Figure 8A**), which expression was negatively correlated with SOX10 (**Figures 8B–K**). The results indicate that SOX10 had no modulation of immune checkpoints.

### Validation of SOX10 expression via immunohistochemical assays and HPA database analysis

**Figure 9:**
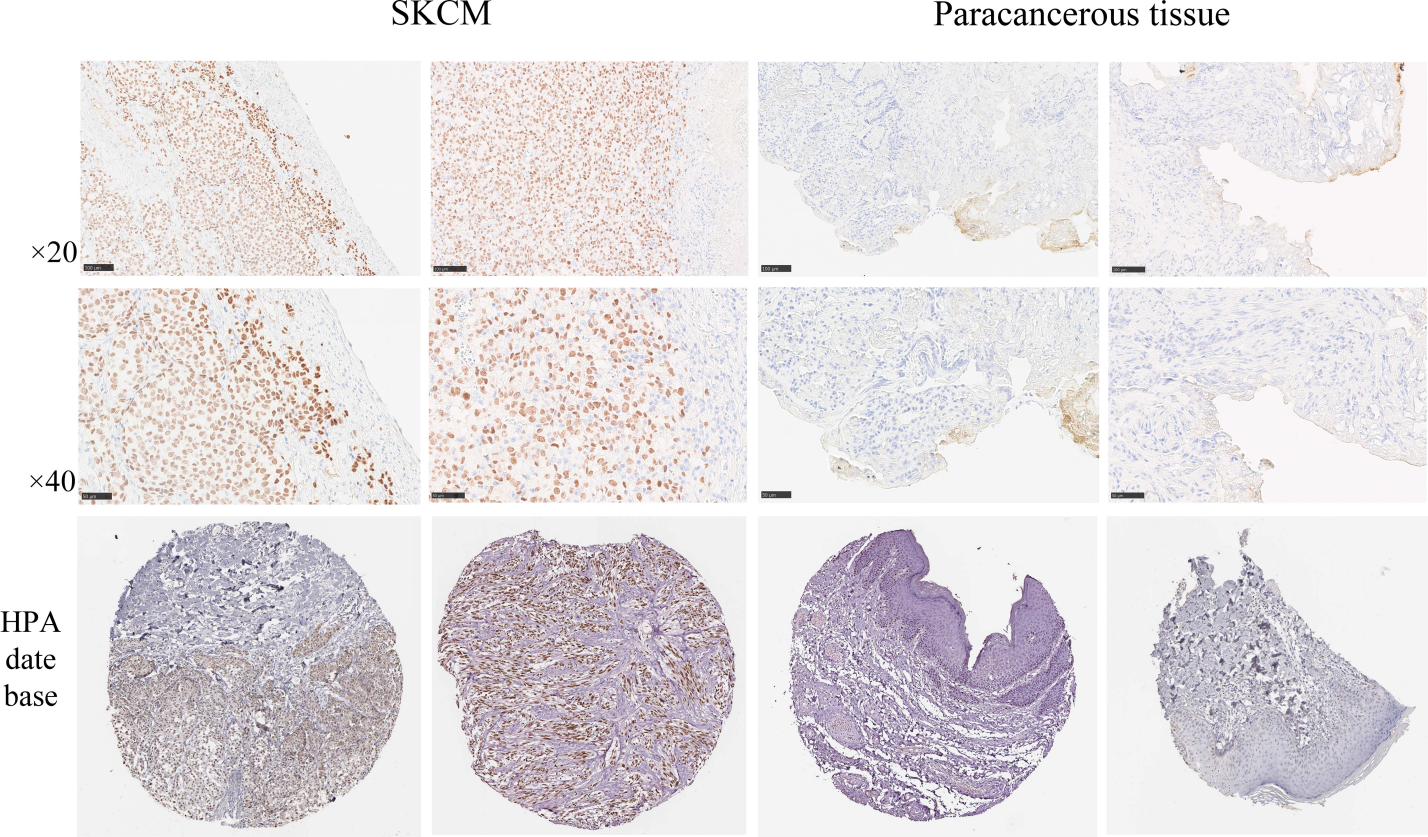
SOX10 with the Human Protein Atlas database and immunohistochemical assays to analyze the protein expression levels. Scale bar = 50 μm.

To ascertain the expression levels of SOX10 in SKCM, we examined the expression of SOX10 in both SKCM tissues and adjacent non-cancerous tissues. Immunohistochemical findings revealed that the expression of SOX10 was significantly higher in SKCM tissues compared to the adjacent tissues. This observation was further corroborated by supplementary data from the HPA database, which demonstrated widespread expression of SOX10 in the perinuclear region (**Figure 9**).

### Comparison of tumor volume and mouse survival

**Figure 10:**
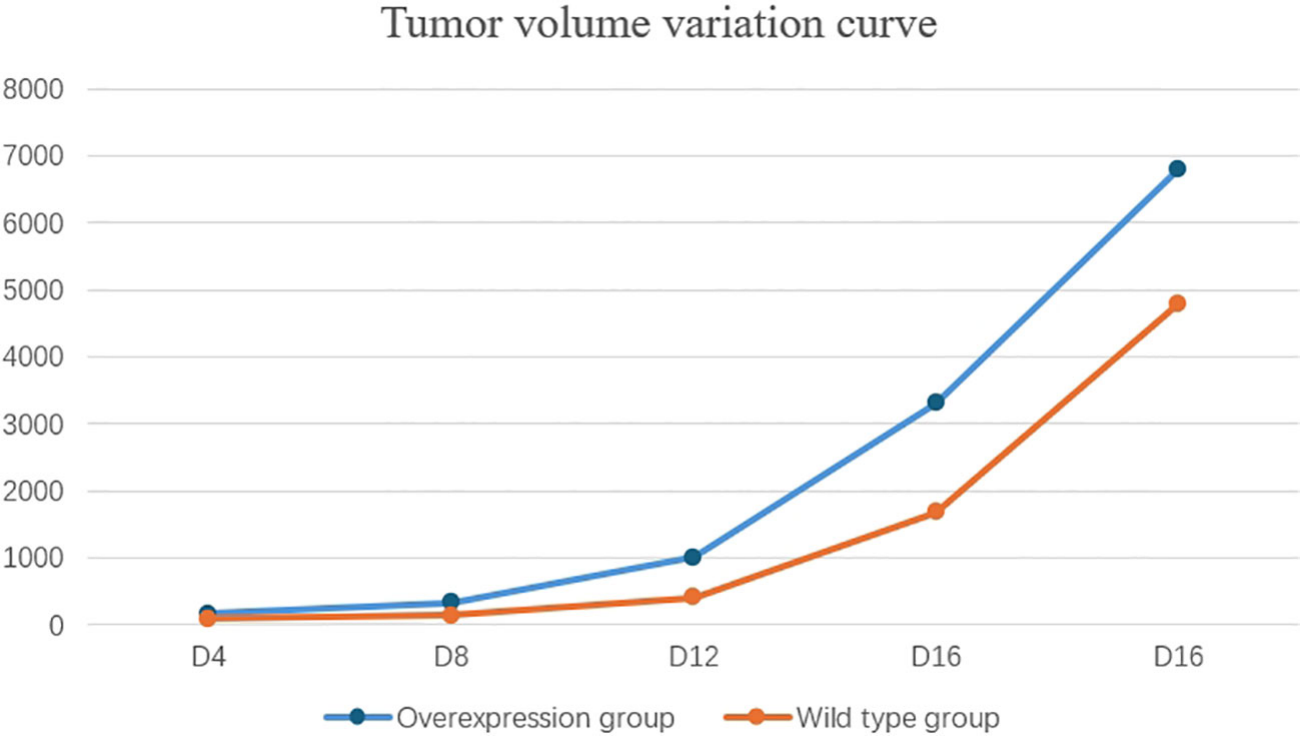
The tumor growth rate in the overexpression group was significantly faster than that in the wild-type group.

By the fourth day post-injection (D4), palpable nodules the size of mung beans, indicative of tumor formation, were detected on the backs of mice in the experimental group. Throughout the growth period, tumor ulceration and scabbing were observed, accompanied by prominent surrounding vasculature. By the twentieth day (D20), 80% of the mice in the experimental group exhibited lethargy, prompting the cessation of tumor volume monitoring. The overexpression group demonstrated a significantly faster tumor growth rate compared to the wild-type group, with statistical differences noted between the two groups (D4, P=0.003; D8, P<0.001; D12, P<0.001; D16, P<0.001; D20, P<0.001) (**Table 4**, **Figure 10**).

**Table 4:** Comparison of tumor volume parameters between two groups of mice (Mean ± SD).

Time	Overexpression group	Wild-type group	P
D4	169.42 ± 50.68	83.98 ± 12.26	0.003
D8	336.77 ± 50.46	145.92 ± 22.21	<0.001
D12	1007.95 ± 86.97	412.09 ± 44.19	<0.001
D16	3305.62 ± 379.11	1677.63 ± 115.78	<0.001
D20	6805.71 ± 520.73	4794.86 ± 630.54	<0.001

## Discussion

A rising number of evidence have confirmed that SOX10 promotes the development of SKCM, Olga Shakhova et al. have found that in human patients, virtually all melanomas are SOX10 positive (23). SOX10 is a key regulator of melanoma progression and promotes a melanocytic/differentiated state (24). Knockdown of SOX10 inhibits melanoma cell proliferation and suppresses tumor formation *in vivo (* 25). Similarly, SOX10 deletion inhibited the growth of melanoma cell lines *in vivo (* 26). Our study found that SOX10 is specifically highly expressed in SKCM. Enrichment analysis was conducted on the co-expressed genes of SOX10 and the differentially expressed genes in tumor tissues of low- and high- SOX10 expression groups. The results showed that high expression of SOX10 could promote oxidative phosphorylation and amino acid metabolism related pathways which accelerated energy metabolism in tumor cells. Meanwhile, SOX10 may promote the formation of an immunosuppressive microenvironment by inhibiting immune-related signaling pathways such as antigen-antibody binding. It has been reported that SOX10 reduced the immunogenicity of melanoma cells by activating the IRF4-IRF1 axis (18). These results could provide a better understanding of the potential mechanisms underlying the pro-tumorigenic effect of SOX10.

High expression of SOX10 was an independent risk factor for SKCM and significantly associated with poor prognosis. Furthermore, SOX10 could be used as a diagnostic indicator for SKCM and various subgroup patients, including pathological stages I&II, age>60, and breslow depth>3 through univariate logistic regression and ROC curves. Significantly elevated serum SOX10 concentrations in SKCM patients allowed for dynamic monitoring of patients’ response to treatment, suggesting that it may serve as a timely hematological blood indicator for evaluating patient outcomes (13). High levels of SOX10 can be detected in the peripheral blood of some low-risk or non-proliferative high-risk SKCM patients, and there are no signs of disease recurrence. In addition, due to high sensitivity and specificity, SOX10 can also serve as a diagnostic marker for sentinel lymph node metastatic melanoma (15). In summary, SOX10 could be a new valuable biomarker for SKCM. It is worth noting that high expression of SOX10 suggests poor prognosis in SKCM. Recently, it has been shown that SOX10/SOX11/MITF (melanocyte inducing transcription factor) can be taken as a therapeutic indicator for SKCM (16). Further screening of SOX10 co expressed genes by LASSO, we construct a prognostic model combined with the expression levels of SOX10, OCA2, and TRAT1 in SKCM, which predict patient OS for 1, 3, and 5 years. The establishment of the nomogram prediction model further confirmed the predictive effect of SOX10 expression on prognosis. The calibration chart showed optimal agreement between the predictions of the nomogram associated with SOX10 and the actual observations of 1-year, 3-year, and 5-year OS probabilities. Through oncogenic analysis, this study discovered that SOX10 expression is significantly upregulated in SKCM and shows specificity (**Figure 1B**). As indicated by the K-M survival curve, SOX10 expression is associated with the onset and progression of SKCM. Consequently, developing a prognostic model based on SOX10 holds significant clinical importance and valuable scientific research potential. Firstly, significant differences in SOX10 expression levels between melanoma and normal skin tissue suggest that SOX10 could act as a marker for differential diagnosis. Secondly, analysis of SOX10 expression also serves as a diagnostic marker for tumors of melanocytic origin. Compared to S-100 and HMB45, SOX10 demonstrates a higher positivity rate (27). Additionally, the role of SOX10 in melanoma drug resistance has been explored. Studies have shown that SOX10 contributes to melanoma’s adaptive resistance to RAF inhibitors via the ERK1/2/SOX10/FOXD3/ERBB3 signaling pathway (28).The imperative for developing a prognostic model based on SOX10 is to enhance melanoma patients’ survival rates and refine treatment strategies. The SKCM prognostic model associated with SOX10 established in this study demonstrates high accuracy. We discovered that SOX10 influences immunoinfiltration in SKCM. Research indicates that predictive models, constructed from gene expression disparities linked to SOX10, and risk scores assessing the tumor immune microenvironment, which can guide clinical immunotherapy (29). Our prognostic model offers the advantage of delivering more precise personalized treatments, pinpointing potential beneficiaries for immunotherapy or targeted therapy, and may identify new therapeutic targets, such as OCA2 featured in the model, a crucial pigment gene in mammals involved in melanin synthesis. Its single nucleotide polymorphism (SNP) rs4778137 strongly correlates with pathological complete remission (pCR) in neoadjuvant chemotherapy for breast cancer and the prognosis for patients with early-stage breast cancer (30, 31). The establishment and verification of the SOX10 prognostic model offer a novel biomarker for clinical use, aiming to enhance patient treatment outcomes and quality of life. However, limited by the number of clinical samples and the lack of evidence-based medicine, the diagnostic and prognostic value of SOX10 in SKCM needs to be further validated in more clinical practice.

The complex interactions between multiple immune cells construct the tumor immune microenvironment (TME) and participate in the occurrence and development of tumors. Neutrophils, as important effector cells in the innate immune system, which recruit in TME and play regulatory roles in multiple stages of tumor development (32). Research has shown that neutrophils can exert cytotoxic effects on tumor cells by secreting ROS, thereby inhibiting the progression of SKCM (33, 34). M1 macrophages can also recruit Th1 cells by secreting various chemokines which can killing tumor cells and promoting adaptive immune response (35). Additionally, CD8^+^T cells can recognize tumor cell antigenic epitopes effectively through antigen presentation effects, which carry out effective killing of tumors (36). Gloger et al (37) isolated and identified over 200 tumor associated antigen epitopes from 5 SKCM cell lines. The current clinical research on T-cell receptor engineered T cells (TCR-T) for the treatment of SKCM has shown significant prolongation of patient survival which confirming the therapeutic potential of TCR-T in SKCM (38). Research indicated that SOX9 can inhibit dendritic cells from infiltrating tumor tissue. Consequently, the absence of antigen presentation indirectly suppresses the infiltration and activity of CD8^+^ T cells and NK cells (39). SOX10 and SOX9 are functionally antagonistic regulators of melanoma development (40). This study discovered that SOX10 suppresses the infiltration of most immune cells in melanoma, with the exception of NK cells. Hence, we propose that SOX10 plays a positive role in the infiltration of NK cells into melanoma tissue. However, there is currently a lack of evidence for the regulation between SOX10 and NK cells, and their roles in tumor initiation and progression. Therefore, further research is required to explore this phenomenon. In short, we founded that high expression of SOX10 inhibits immune cell infiltration in SKCM. However, there is a negative correlation between the expression of SOX10 and various immune checkpoints in SKCM, although the expression of each immune checkpoint is upregulated in tumors. These results reveals that SOX10 may not contribute to the development and progression of melanoma by influencing the expression of immune checkpoints like PD-1/PDL1. Studies have demonstrated that SOX10 suppresses the expression of PD-L1 through the regulation of the IRF4-IRF1 axis (18).This finding aligns with the results of this study. However, research indicated that IRF1 can promote the expression of PD-L1 and enhance tumor cells’ ability to evade recognition and elimination by T cells (41). Similarly, Sasaki proposed that overexpression of SOX10 significantly upregulated the expression of PD-L1 in tumor cells (42).Therefore, the relationship between SOX10 and immune checkpoints requires further exploration in future studies. In addition, the GSEA analysis results suggest that high expression of SOX10 may exert a pro tumor effect by inhibiting the interferon gamma (IFN-γ) response and JAK/STAT signaling pathway. JAK-STAT is a key signaling pathway that promotes cell growth and development, which can be regulated by various cytokines such as interferon, interleukin, and colony stimulating factor. It plays an important role in the occurrence and development of various cancers (43–45). JAK mutations can block IFN-γ signal transduction to mediate tumor immune escape and inhibits PD-1/PD-L1 related immunotherapeutic effects (46, 47). In addition, inhibiting the activation of the JAK-STAT signaling pathway could promote the resistance of melanoma cell lines to interferon (48, 49). Collectively, SOX10 may inhibit the killing effect of immune cells on SKCM and promote tumor immune escape by inhibiting immune cell infiltration and IFN-γ/JAK/STAT signaling pathway. Therefore, the immune escape mechanism of SKCM needs to be further explored.

## Conclusions

In conclusion, this research reveals for the first time that SOX10 up-regulation is associated with poor prognosis in SKCM. Additionally, SOX10 probably attenuated the tumor killing effect of T cells by blocking the T cell-mediated interferon-γ and JASK/STAT signaling pathways through inhibiting immune infiltration.

## Data Availability

The datasets presented in this study can be found in online repositories. The names of the repository/repositories and accession number(s) can be found in the article/**Supplementary Material**.
