# Supplementary material for: High expression of SOX10 is correlated with poor prognosis and immune infiltrates in skin cutaneous melanoma
Source: Front Oncol. 2025 Apr 24;15:1444670. doi: 10.3389/fonc.2025.1444670 (PMC12058902; doi:10.3389/fonc.2025.1444670)
Supplement: Supplementary file 2 [file DataSheet2.docx]

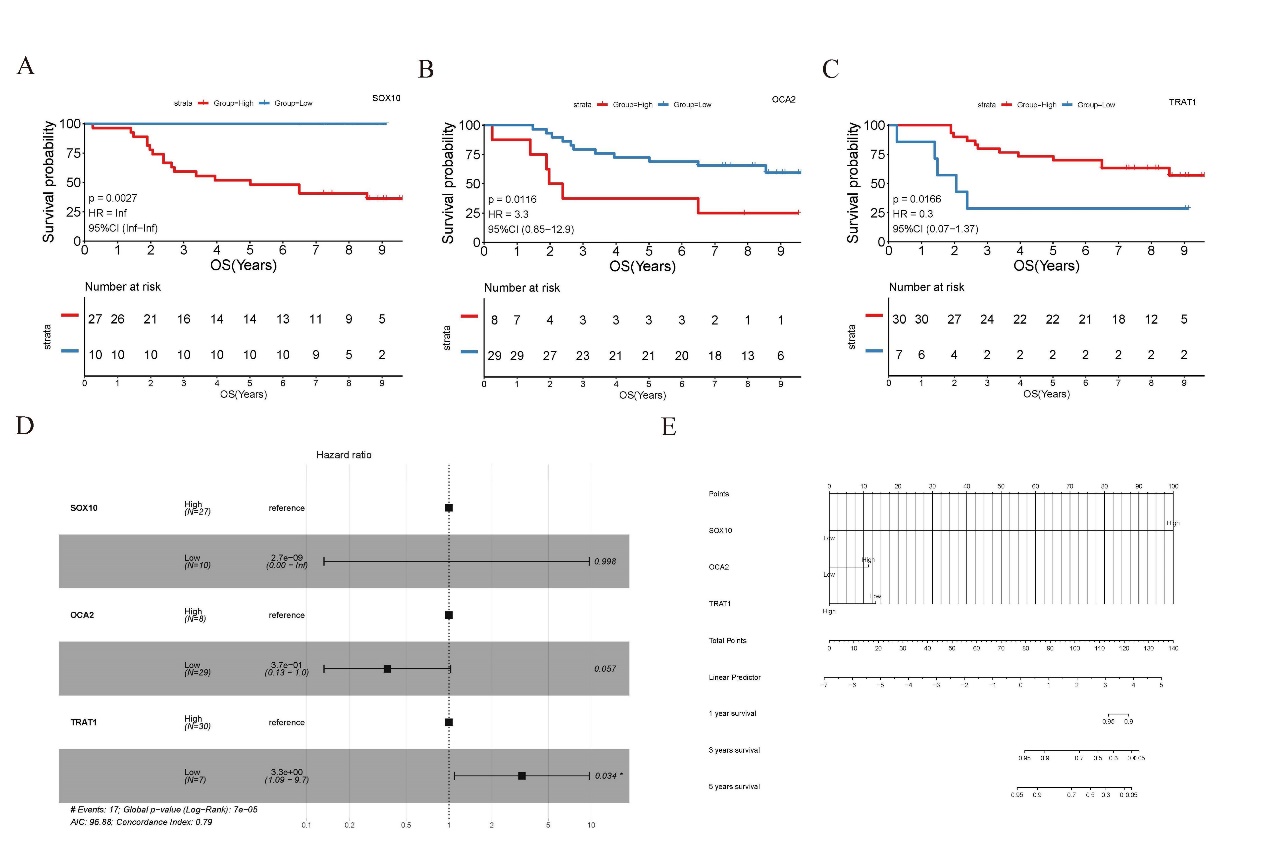


**Figure Supplementary 1.** **Verification cohort of GSE98394.** A-C: KM curves with SOX10、OCA2 and TRAT1 expression shows the survival probability of the validation cohort GSE98394; D: Forest plot of SOX10、OCA2 and TRAT1; E: prognostic risk models for high- and low-risk groups is validated in GSE98394.

Explanation of the Results of Supplementary Material：

We retrieved the dataset (GSE98394) from the GEO database to further validate the model's accuracy. In the GSE98394 dataset, significant differences were observed in the overall survival (OS) rates between high and low risk score groups for SOX10, OCA2, and TRAT1, P= 0.0027，P=0.0116，P=0.0166 **(Figure.S1A-C**), The results showed that OCA2 and TRAT1 were negative prognostic indicators and positive prognostic indicators（**Figure.S1D**）Given the effects of OCA2 and TRAT1, a nomogram was constructed including expression of SOX10, OCA2 and TRAT1. The 1-, 3-, 5-year survival probability was determined by drawing a vertical line downward on the total point axis suggesting the probability of 1-, 3- and 5-year > 50%( **Figure.S 1E**). The predictive model demonstrates high accuracy in the validation cohort, and its trends align with the predictions derived from the TCGA database. In conclusion, this model can reliably estimate the overall survival of SKCM patients over 1-, 3- and 5-year.
